# Systematic Analysis of Experimental Phenotype Data Reveals Gene Functions

**DOI:** 10.1371/journal.pone.0060847

**Published:** 2013-04-16

**Authors:** Robert Hoehndorf, Nigel W. Hardy, David Osumi-Sutherland, Susan Tweedie, Paul N. Schofield, Georgios V. Gkoutos

**Affiliations:** 1 Department of Physiology, Development and Neuroscience, University of Cambridge, Cambridge, United Kingdom; 2 Department of Computer Science, University of Aberystwyth, Old College, Aberystwyth, United Kingdom; 3 Wellcome Trust/Cancer Research United Kingdom Gurdon Institute, University of Cambridge, Cambridge, United Kingdom; 4 Department of Genetics, University of Cambridge, Cambridge, United Kingdom; The Scripps Research Institute, United States of America

## Abstract

High-throughput phenotyping projects in model organisms have the potential to improve our understanding of gene functions and their role in living organisms. We have developed a computational, knowledge-based approach to automatically infer gene functions from phenotypic manifestations and applied this approach to yeast (*Saccharomyces cerevisiae*), nematode worm (*Caenorhabditis elegans*), zebrafish (*Danio rerio*), fruitfly (*Drosophila melanogaster*) and mouse (*Mus musculus*) phenotypes. Our approach is based on the assumption that, if a mutation in a gene 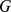 leads to a phenotypic abnormality in a process 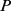, then 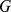 must have been involved in 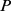, either directly or indirectly. We systematically analyze recorded phenotypes in animal models using the formal definitions created for phenotype ontologies. We evaluate the validity of the inferred functions manually and by demonstrating a significant improvement in predicting genetic interactions and protein-protein interactions based on functional similarity. Our knowledge-based approach is generally applicable to phenotypes recorded in model organism databases, including phenotypes from large-scale, high throughput community projects whose primary mode of dissemination is direct publication on-line rather than in the literature.

## Introduction

The functional annotation of genes and their products using the Gene Ontology (GO) [1] has been essential to the impact of recent advances in the biomedical sciences arising from the explosion of genome sequences now becoming available. The majority of GO annotations are manually asserted by trained experts based on literature evidence. Recently, large-scale community projects, using forward and reverse genetics, as well as pan-genomic phenotyping efforts, such as the International Mouse Phenotyping Consortium (IMPC) [2] and the Zebrafish mutation project [3], have begun the systematic phenotyping of animal model mutants. Such efforts have a huge potential to provide novel insights into gene functions and their roles in disease. However, data resulting from high-throughput phenotyping efforts are not immediately reported in the literature, and gene functions are not readily inferred from phenotype data. Here, we present a novel method to automatically infer functions of gene products from phenotype data. Our method is applicable to both manually assigned phenotype annotations and those resulting from electronically reported, high-throughput phenotype experiments.

GO annotations are comprised of a gene product, a term that represents molecular function, biological process or a cellular component, the literature reference for the assignment, and an evidence code that indicates how the annotation was derived. Annotations of genes are maintained in model organism databases and the GO annotation (GOA) database [4] which now covers over 160,000 taxa and more than 32 million annotations. The strategies for annotating genes and proteins range from explicit expert manual curation of the literature, through electronic inference based on orthology, protein-protein or genetic interactions, to inference of functions based on protein family relations. GO annotations for humans currently comprise 353,102 annotations for 45,364 proteins (based on GOA version 115, accessed December 2 2012), of which approximately half are manually curated, the remainder being derived electronically. In the mouse, there are 25,437 protein-coding genes with GO annotation (both electronic and manual), and 9,990 proteins with experimentally-derived annotations. The scale of the annotation task and the speed with which new genomes are becoming available has necessitated the development of automated and semi-automated annotation strategies to maintain significant coverage, and several automated function annotation methods have gained importance in recent years [5], [6]. However, phenotype data has not yet been employed on a large scale as a source of high-quality electronic annotations.

Animal model phenotypes are commonly characterized using a species-specific phenotype ontology, many of which are based on the Entity/Quality (EQ) framework [7]. While phenotype data has traditionally been gathered through literature curation, the results of high-throughput phenotyping are commonly made available directly in research databases without an associated report in the literature. These results are therefore not available through literature curation or inference of gene functions from phenotypes by trained curators. A systematic exploitation of phenotype data to identify gene functions requires a computational approach that can assign functions to genes based on the recorded phenotypes. Such an approach is applicable to manual curation of phenotype data from the literature as well as data derived from high-throughput phenotyping efforts.

The main challenge in designing such an approach is to relate phenotype observations in mutagenesis experiments, which are characterized using terms in species-specific phenotype ontologies, systematically to gene functions, which are described using the Gene Ontology [1]. We have developed a method that employs the logical definitions of terms in phenotype ontologies [8] and infers functions of genes and gene products from phenotype statements. Our method relies on the assumption that, if a mutation of a gene 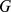 results in a phenotypic manifestation that affects a GO process or function 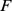, then 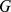 must have been involved in the function 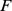. For example, if a phenotypic observation of a targeted gene knockout mouse mutant is *abnormal B cell apoptosis*, then the mutation must have occurred in a gene that is involved in *B cell apoptosis*. We apply our approach to yeast (*Saccharomyces cerevisiae*) [9], fly (*Drosophila melanogaster*) [10], fish (*Danio rerio*) [11], worm (*Caenorhabditis elegans*) [12] and mouse (*Mus musculus*) [13] phenotype data and identify several thousand novel associations between genes and their functions.

The quality of gene function annotations is often evaluated based on inter-annotator agreement [14], [15] or a gold standard [16]. However, for novel functions that either have not yet been extensively studied or have not yet been reported in the literature, these approaches are not readily applicable. Therefore, in order to assess the quality of our inferred annotations, we applied the phenotypically inferred functions to the task of predicting known genetic and protein-protein interactions based on functional similarity over GO biological functions [17]–[19]. We use the GO annotations of gene products available from the various model organism databases as well as the GOA database [4] as baseline for predicting genetic interactions and PPIs, and compare the results to manually curated and experimentally validated genetic interactions and PPIs using Receiver Operating Characteristic (ROC) analysis [20]. We then perform the same analysis again, adding our inferred GO annotations to the annotations already available from the model organism databases and GOA. For each species, we identify an increase in the performance of predicting known genetic interactions and PPIs when adding the functions we infer, and, in most cases, the increase in performance is significant. When combining the inferred annotations across all five species and predicting genetic interactions and PPIs, we also find a significant increase in ROC AUC (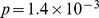 for PPIs, 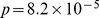 for genetic interactions, 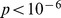 for PPIs from STRING, 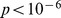 for BioGRID interactions; one-tailed t-test).

The inferred functions and our analysis code are freely available on http://phenotype2go.googlecode.com.

## Materials and Methods

### Predicting Functions from Phenotypes

Table 1 provides an overview of the datasets and resources used in this work. Phenotypes in the yeast, fly, worm and mouse phenotype ontologies were manually defined [8] using the PATO framework [7], while fish phenotypes are directly annotated using PATO. According to PATO, a phenotype statement can be decomposed into one or more *entities* (E) that are affected in a phenotype and a *quality* (Q) that determines how the entity is affected. Table 2 shows the number and completeness of the phenotype definitions for yeast, fly, worm and mouse phenotypes ontologies.

**Table 1:** Overview over main resources used in this work.

Data	Downloaded from	Downloaded on/Version
GO annotations	Gene Ontology Annotation project [4]	Nov 13, 2012
Genetic interactions	Gene Ontology Annotation project	Nov 13, 2012
Protein-protein interactions	Gene Ontology Annotation project	Nov 13, 2012
Protein-protein interactions	STRING [21]	Dec 25, 2012/v9.0
Protein-protein and genetic interactions	BioGRID [22]	Feb 20, 2013/v3.2.97
Phenotype data	Model organism databases (MGI, SGD, ZFIN, FlyBase, WormBase)	Nov 11, 2012
Phenotype ontologies	OBO Foundry website	Nov 13, 2012
Gene Ontology	Gene Ontology website	Nov 13, 2012
Mammalian Phenotype Ontology: formal definitions	https://phenotype-ontologies.googlecode.com/svn/trunk/src/ontology/mp/mp-equivalence-axioms.obo	Nov 13, 2012
Human Phenotype Ontology: formal definitions	https://phenotype-ontologies.googlecode.com/svn/trunk/src/ontology/hp/hp-equivalence-axioms.obo	Nov 13, 2012
Worm Phenotype Ontology: formal definitions	http://obo.cvs.sourceforge.net/checkout/obo/obo/ontology/phenotype/worm_phenotype_xp.obo	Nov 13, 2012
Ascomycete Phenotype Ontology: formal definitions	http://obo.cvs.sourceforge.net/viewvc/obo/obo/ontology/phenotype/yeast_phenotype_xp.obo	Nov 13, 2012
Flybase Controlled Vocabulary: formal definitions	http://code.google.com/p/phenomeblast/source/browse/trunk/phenotypeontology/obo/fly_xp.obo	Nov 13, 2012

The phenotype data resulting from single gene mutations and genetic interaction data for *Drosophilae*, *Mus musculus*, *Saccharomyces cerevisiae*, *Caenorhabditis elegans* and *Danio rerio* were downloaded from *FlyBase* [10], *Mouse Genome Informatics* [13], *Saccharomyces* Genome Database [9], *WormBase* [12], and *Zebrafish Information Network* (ZFIN) database [11] respectively. All phenotype data was downloaded on 11 Nov 2012. GO annotations were downloaded from the GOA website on 13 Nov 2012. The ontologies as well as the phenotype definitions were obtained from the OBO Foundry website on 13 Nov 2012. For zebrafish, we downloaded the files Morpholinos.txt and genotype_features.txt on 02 Oct 2012 from the ZFIN website. We use these files to map ZFIN genotype and Morpholino identifiers to ZFIN’s gene identifiers.

**Table 2:** Overview of the completeness of definitions for several phenotype ontologies.

Phenotype ontology	Number of classes in ontology	Number of classes defined
Mammalian Phenotype Ontology	9,241	6,587
Ascomycete phenotype Ontology	329	159
C. elegans phenotype Ontology	2,095	942
FlyBase Controlled Vocabulary	821	743

To identify gene functions from phenotypes, we first identified the quality and the entity of the animal model’s phenotype annotation and then identified the genes that have been mutated in the animal model. If the *entity* that is part of the phenotype statement is based on the GO, we assign that GO term as a function to the gene that has been mutated in the animal model. For example, the mouse model *Vgf* 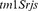 (MGI:2179681), a targeted mutation of the *Vgf* gene, is characterized by a phenotype *lactation failure* (MP:0010249). *Lactation failure* is decomposed into the entity *lactation* (GO:0007595) and the quality *lacking processual parts* (PATO:0001558). Since *lactation* is impaired in the phenotype resulting from a mutation in *Vgf*, we infer that *Vgf* must be involved in *lactation*.

### Predicting Interactions

We evaluate the inferred gene functions by applying them to the prediction of genetic interactions and PPIs. To obtain the genetic interactions and PPIs, we use the GO annotation files and identify GO annotations with the IGI (inferred from genetic interaction) and IPI (inferred from protein interaction) evidence codes. The GO annotations contain as additional evidence the interaction partner from which the annotation has been inferred, and we use this pair as a genetic interaction or PPI (depending on the evidence code). Since the use of interactions contained in the GO annotation files may introduce a bias when predicting interactions based on GO annotation similarity, we further use known PPIs from the STRING database [21] and interactions from the BioGRID database [22] to provide additional independent verification datasets.

We then filter the sets of interaction data from the model organism databases and remove the interaction pairs for which we have not inferred a novel function (i.e., if 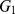 and 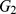 interact but we were not able to infer a novel function for 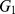 or 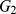, we remove this pair from the interaction data set). For each species for which we infer novel functions, we then perform a pairwise computation of functional similarity between genes. To calculate the similarity between two sets of GO annotations, we used the Jaccard index as a measure of semantic similarity. If a gene 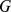 has the GO terms 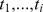 as annotations, we generate the set 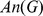 as the smallest set that contains 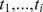 and is closed against superclasses (i.e., if 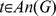, and 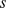 is a superclass of 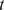, then 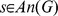). We then define the similarity between the genes 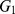 and 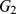 as:
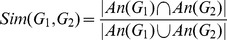
(1)


While a large number of different semantic similarity measures exists [19], we chose to apply the Jaccard index as it does not rely on information content to determine similarity. While similarity measures that incorporate the information content of an ontology term commonly provide better performance than measures that do not use information content [19], they may also introduce a bias when comparing the results of an analysis performed on multiple independent datasets. To ensure comparable results across all species we analyze, we used the Jaccard index without any weights based on information content.

As a result of applying this similarity measure, we obtain, for each gene, a functional similarity value to all other genes. For each gene 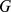, we then rank this similarity list so that the gene that is functionally most similar to 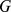 is on rank 1 and the least similar on the last rank. Using the genetic interaction and PPI datasets as positive instances and all other pairs as negative, we then predict genetic interactions and PPIs based on functional similarity. We measure the success using an analysis of the receiver operating characteristic (ROC) curve and determine the area under the ROC curve (ROC AUC) [20]. A ROC curve is a plot of the true positive rate as a function of the false positive rate and can be used to visualize the quality of the predictions. The ROC AUC is a quantitative measure of the classifiers performance: a ROC AUC of 0.5 indicates a random classifier (i.e., the true positive rate increases proportional to the false positive rate), while a ROC AUC of 1 indicates a perfect classifier (i.e., all true positive instances are placed on the first rank, while the true negative instances are all ranked lower).

In the absence of a large set of true negative examples of PPIs or genetic interactions, we make the assumption that interactions that are not present in our evaluation datasets are negative instances. As a consequence, our true positive rate is lower than the one that we would obtain when treating only validated negative interactions as negative examples. Furthermore, the resulting ROC AUCs are also lower than the ones we would achieve with validated negative examples of interactions. Since we use the same positive and negative instances (for each species) to perform our comparative evaluation of current and inferred GO functions, this assumption will not affect the validity of our results.

## Results and Discussion

### Prediction of Gene Functions

Applying our method, we extract 1,409 novel associations between genes and their functions for zebrafish, 12,483 for yeast, 1,057 for fruitfly, 3,885 for worm and 14,013 for mouse, using only the GO annotations with manually created evidence for comparison (evidence codes *Inferred from Experiment* (EXP), *Inferred from Direct Assay* (IDA), *Inferred from Physical Interaction* (IPI), *Inferred from Mutant Phenotype* (IMP), *Inferred from Genetic Interaction* (IGI) and *Inferred from Expression Pattern* (IEP)). We evaluate the quality of the inferred functions both manually and by applying them for predicting known genetic interactions and PPIs. First, we randomly selected 20 annotations from each species and examined scientific papers in which the gene and the resulting phenotypes are discussed. We find that the annotations that we generate are biologically valid if the phenotype annotations, and the formal definitions of the terms that are used to described them, are accurate. For example, we infer that the mouse gene *Efnb2* (MGI:105097) is involved in *cloacal septation* (GO:0060197), a function that has previously been reported in the literature [23] but which has not yet been added as a GO annotation of *Efnb2*. In several cases, the annotations we generate are too general, i.e., a biologist would be able to infer a more specific function from the described experiment; nevertheless, even the general annotations are valid and may provide useful information about a gene’s function. The detailed manual evaluation results, including references to the manuscripts that support the novel annotation, are included as supplementary material.

**Figure 1:**
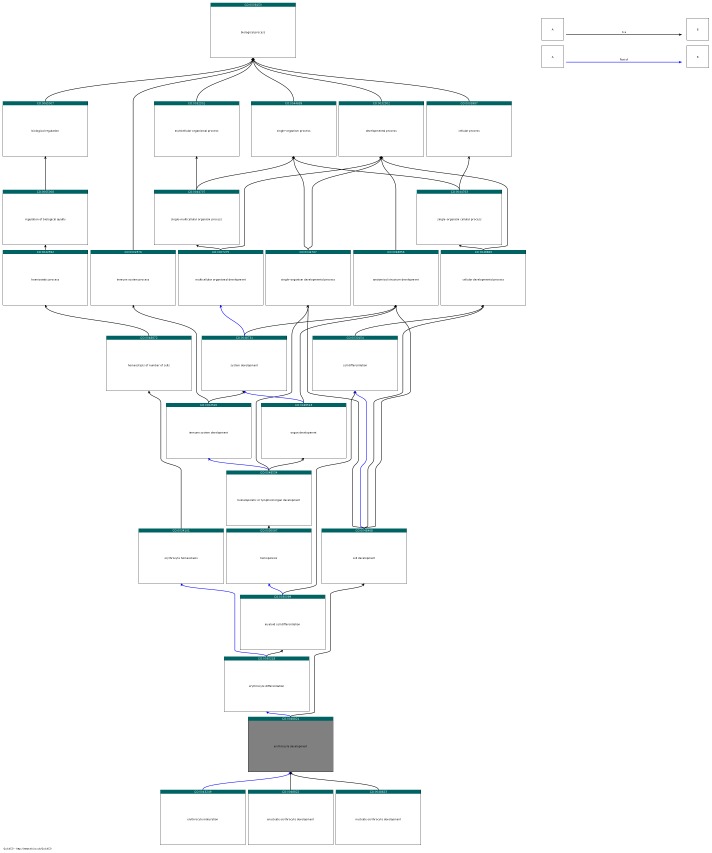
Sub-graph of the GO hierarchy displaying ancestors and children of *erythrocyte development*. The figure has been created using the QuickGO browser [**34**].

We also found evidence in some systems for an improvement in annotation granularity. Taking the novel annotations in the mouse genome to *erythrocyte development* (GO:0048821), we manually examined the underlying phenotype evidence in MGI for the new assertions, together with the existing GO process and function annotations. Of 77 novel annotations to *erythrocyte development*, 29 genes already had some annotation to *erythrocyte differentiation* (GO:0030218) or regulation of the erythroid or myeloid lineages, with the most common annotation being to the parent term *erythrocyte differentiation*. Some genes were annotated to directional regulation, such as *positive regulation of erythrocyte differentiation* (GO: 0045648), but others were annotated to much more general GO terms such as *myeloid cell differentiation* (GO:00030099). The remaining genes with novel annotations to *erythrocyte development* have no current GO annotation to erythroid lineage processes but mutants show phenotypes affecting erythroid differentiation or development. Table 3 provides an overview over our manual evaluation results for annotations to *erythrocyte development*, and Figure 1 shows the corresponding part of the GO hierarchy.

**Table 3:** Predicted annotations of mouse genes for erythrocyte development (GO:0048821).

MGI ID	Gene	Existing GO annotation	Allele supporting new annotation
MGI:1921354	*Abcb6*		*Abcb6* 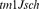
MGI:2158492	*Ahsp*	GO:0030218 erythrocyte differentiation	
MGI:88064	*Ar*		*ArTfm*
MGI:88113	*Atp4a*		*Atp4a* 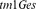
MGI:106190	*Bcl11a*		*Bcl11a* 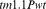
MGI:1338013	*Cbfa2t3*		*Cbfa2t3* 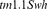
MGI:88316	*Ccne1*		*Ccne1* 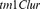
MGI:96617	*Cd47*		*Cd47* 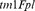
MGI:103198	*Cdc25a*		*Cdc25a* 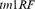
MGI:2384875	*Cdk5rap2*		*Cdk5rap2* 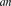
MGI:1344352	*Dido1*		*Dido1* 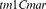
MGI:1329019	*Dnase2a*	GO:0030218 erythrocyte differentiation	
MGI:1330300	*Dyrk3*	GO:0030218 erythrocyte differentiation	
MGI:103012	*E2f4*		*E2f4* 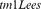
MGI:95295	*Egr1*		*Egr1* 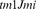
MGI:95401	*Epb4.1*		*Epb4.1* 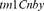
MGI:95407	*Epo*	GO:0030218 erythrocyte differentiation	
MGI:95408	*Epor*		*Epor* 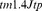
MGI:109336	*Etv6*		*Etv6* 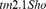
MGI:1351611	*Exoc6*	GO:0030218 erythrocyte differentiation	
MGI:95513	*Fech*	GO:0030218 erythrocyte differentiation	
MGI:95661	*Gata1*	GO:0048821 erythrocyte development	
MGI:95662	*Gata2*	GO:0045648 positive regulation of erythrocyte differentiation	
MGI:1276578	*Gfi1b*	GO:0045646 regulation of erythrocyte differentiation	
MGI:96103	*Hk1*		*Hk1* 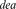
MGI:96109	*Hlx*		*Hlx* 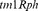
MGI:1098219	*Hmgb3*	GO:0045638 negative regulation of myeloid cell differentiation	
MGI:96187	*Hoxb6*	GO:0034101 erythrocyte homeostasis	
MGI:1342540	*Ikzf1*		*Ikzf1* 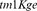
MGI:96560	*Il6st*		*Il6st* 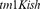
MGI:107357	*Inpp5d*	GO:0045648 positive regulation of erythrocyte differentiation	
MGI:96395	*Irf8*	GO:0030099 myeloid cell differentiation	
MGI:96629	*Jak2*	GO:0030218 erythrocyte differentiation	
MGI:99928	*Jak3*		*Jak3* 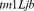
MGI:104813	*Jarid2*		*Jarid2* 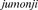
MGI:96677	*Kit*	GO:0030218 erythrocyte differentiation	
MGI:96974	*Kitl*		*Kitl* 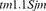
MGI:1354948	*Klf13*	GO:0045647 negative regulation of erythrocyte differentiation	
MGI:96757	*Lcn2*		*Lcn2* 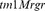
MGI:96785	*Lhx2*		*Lhx2* 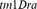
MGI:101789	*Lig1*		*Lig1* 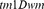
MGI:1346865	*Mapk14*	GO:0045648 positive regulation of erythrocyte differentiation	
MGI:2444881	*Mfsd7b*	GO:0030218 erythrocyte differentiation	
MGI:3619412	*Mir451*		*Mir451* 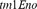
MGI:97249	*Myb*		*Myb* 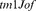
MGI:2442415	*Myst3*	GO:0030099 myeloid cell differentiation	
MGI:1349717	*Ncor1*		*Ncor1* 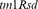
MGI:104741	*Nfkbia*	GO:0045638 negative regulation of myeloid cell differentiation	*Nfkbiatm1.1Kbp*
MGI:97566	*Pgm3*		*Pgm3* 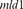
MGI:2385902	*Picalm*		*Picalm* 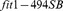
MGI:97583	*Pik3r1*		*Pik3r1* 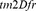
MGI:1201409	*Pknox1*	GO:0030218 erythrocyte differentiation	
MGI:101898	*Pou2f1*		*Pou2f1* 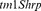
MGI:1927072	*Ppp1r15a*		*Ppp1r15a* 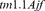
MGI:109486	*Prdx2*		*Prdx2* 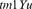
MGI:97806	*Ptpn2*		*Ptpn2* 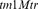
MGI:97810	*ptprc*		*Ptprc* 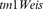
MGI:97874	*Rb1*	GO:0043353 enucleate erythrocyte differentiation	
MGI:103289	*Relb*		*Relb* 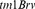
MGI:99852	*Runx1*	GO:0030099 myeloid cell differentiation	*Runx1* 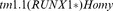
MGI:2146974	*Safb*		*Safb* 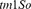
MGI:2445054	*Senp1*	GO:0010724 regulation of definitive erythrocyte differentiation	
MGI:98282	*Sfpi1*	GO:0045646 regulation of erythrocyte differentiation	
MGI:1928761	*slc19a2*		*Slc19a2* 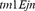
MGI:108392	*Slc20a1*		*Slc20a1* 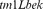
MGI:98354	*Sos1*		*Sos1* 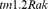
MGI:1277166	*Sp3*	GO:0030218 erythrocyte differentiation	*Sp3tm1Sus*
MGI:98385	*Spna1*		*Spna1* 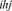
MGI:1915678	*Steap3*		*Steap3* 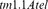
MGI:98480	*Tal1*	GO:0045648 positive regulation of erythrocyte differentiation	
MGI:1196624	*Tcea1*	GO:0030218 erythrocyte differentiation	
MGI:98822	*Tfrc*		*Tfrc* 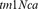
MGI:98729	*Tgfbr2*		*Tgfbr2* 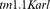
MGI:1920999	*Ttc7*		*Ttc7* 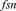
MGI:1270126	*Ulk1*		*Ulk1* 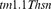
MGI:98917	*Uros*		*Uros* 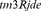
MGI:103223	*Vhl*		*Vhl* 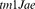
MGI:1095400	*Zfpm1*	GO:0060318 definitive erythrocyte differentiation	

Figure 1 shows the part of the GO hierarchy containing *erythrocyte development*. Of the 77 genes predicted by phenotypic analysis to annotate to erythrocyte development, 29 already had some relevant annotation, shown in column 3. Relevant annotation was taken to be any child class of myeloid cell differentiation (GO:0030099), or erythrocyte homeostasis (GO:0034101), thereby including as many levels of granularity as possible in order to compensate for possible curator decisions to annotate more generally. The remaining 48 genes had no existing annotations to any of these classes. In many cases, multiple genotypes provided evidence for the novel annotation; an example allele is shown in column 4. Phenotype annotations to *abnormal erythropoiesis* (MP:0000245) or its subclasses were counted as evidence. Whilst close curation of the phenotypic evidence may suggest that annotation to a parent of *erythrocyte development* is more appropriate, in all cases the evidence indicated that annotation to the neighbourhood of this class was correct but missing.

**Figure 2:**
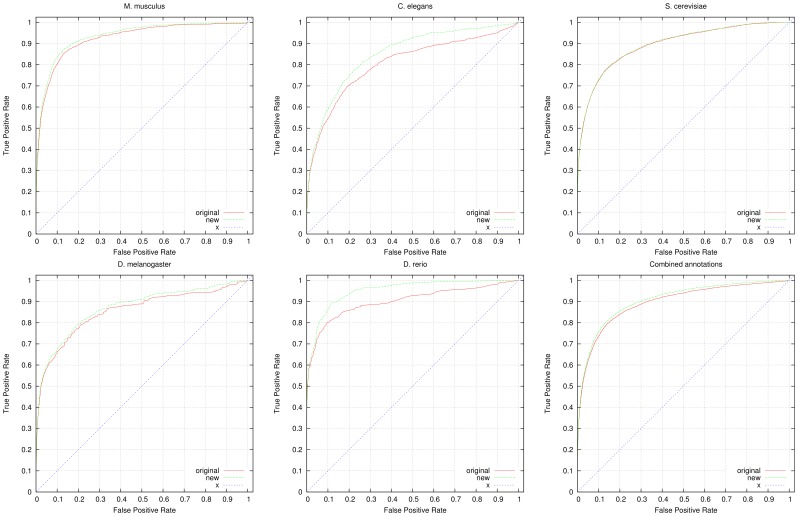
ROC curves for predicting genetic interactions. We compare the performance of predicting genetic interactions using all available annotations from GOA (labeled “original” in the graphs) and using GOA’s annotations combined with our inferred functions (labeled “new” in the graphs). For the evaluation, we used 4,061 genetic interactions in yeast, 783 interactions in fly, 169 interactions in fish, 3,970 interactions in mouse and 893 interactions in worm. We also show the ROC curve resulting from the combined annotations and interactions in all five species. All ROC curves include the line of no-discrimination (labeled “x” in the graphs). The detailed evaluation results are provided as supplementary material.

**Figure 3:**
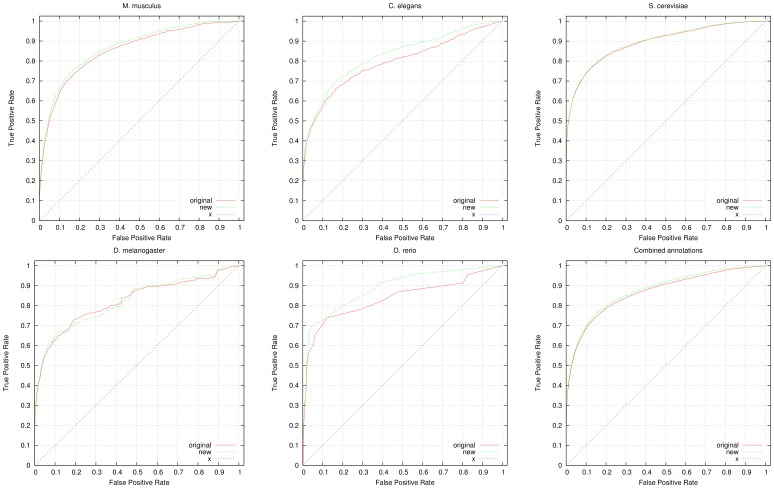
ROC curves for prediction of protein-protein interactions. We compare the performance of predicting protein-protein interactions using all available annotations from GOA (labeled “original” in the graphs) and using GOA’s annotations combined with our inferred functions (labeled “new” in the graphs). For the evaluation, we used 4,834 genetic interactions in yeast, 500 interactions in fly, 23 interactions in fish, 3,152 interactions in mouse and 765 interactions in worm. We also show the ROC curve resulting from the combined annotations and interactions in all five species. All ROC curves include the line of non-discrimination (labeled “x” in the graphs). The detailed evaluation results are provided as supplementary material.

**Figure 4:**
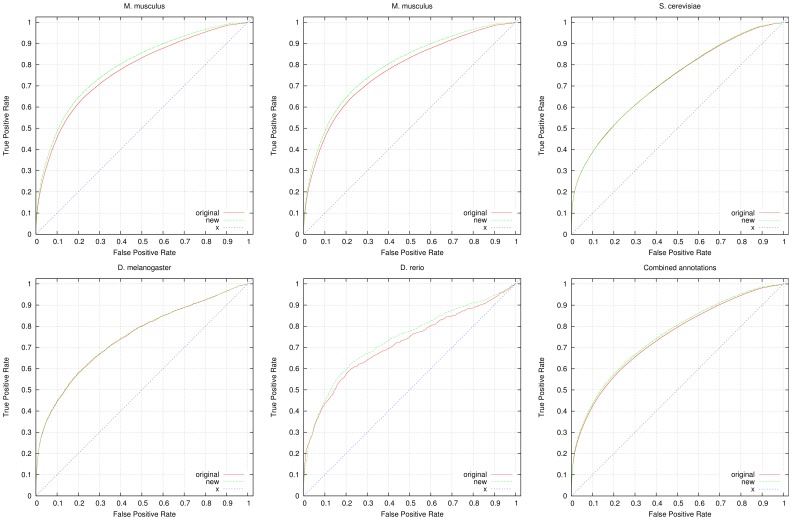
ROC curves for prediction of STRING [**21**] protein-protein interactions. We compare the performance of predicting STRING’s protein-protein interactions using all available annotations from GOA (labeled “original” in the graphs) and using GOA’s annotations combined with our inferred functions (labeled “new” in the graphs). For the evaluation, we used 73,245 interactions in yeast, 4,422 interactions in fly, 1,085 interactions in fish, 42,322 interactions in mouse and 11,517 interactions in worm. We also show the ROC curve resulting from the combined annotations and interactions in all five species. All ROC curves include the line of non-discrimination (labeled “x” in the graphs). The detailed evaluation results are provided as supplementary material.

**Figure 5:**
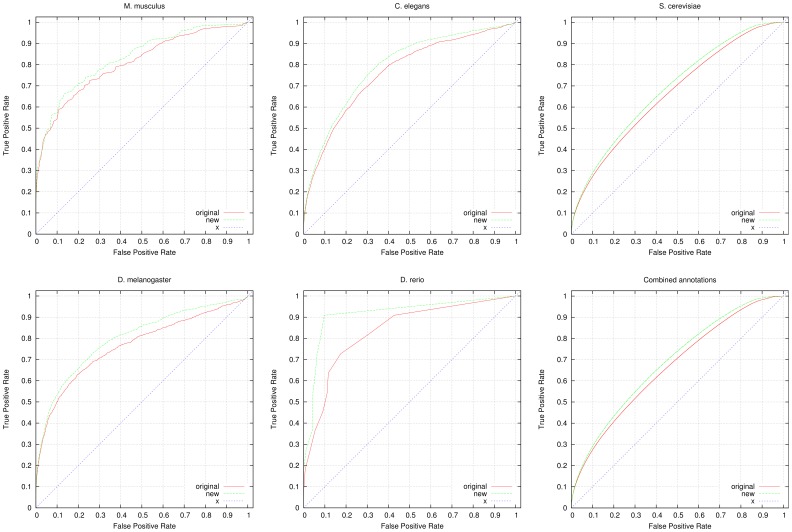
ROC curves for prediction of BioGRID [**22**] protein-protein and genetic interactions. We compare the performance of predicting BioGRID’s interactions using all available annotations from GOA (labeled “original” in the graphs) and using GOA’s annotations combined with our inferred functions (labeled “new” in the graphs). For the evaluation, we used 172,547 interactions in yeast, 1,335 interactions in fly, 11 interactions in fish, 124 interactions in mouse and 2,261 interactions in worm. We also show the ROC curve resulting from the combined annotations and interactions in all five species. All ROC curves include the line of non-discrimination (labeled “x” in the graphs). The detailed evaluation results are provided as supplementary material.

To further evaluate the predicted annotations, we quantify their impact on predicting known genetic interactions and PPIs. For this purpose, we applied a measure of functional similarity between genes (see Materials and Methods section) and rank genes based on their similarity. For evaluating the functional similarity, we use *all* GO annotations available for a gene, including electronically inferred annotations. We then use datasets of genetic interactions and PPIs as a gold standard. We obtain the interactions from the GO annotations tagged with the *IGI* (inferred from genetic interaction) and *IPI* (inferred from protein interaction) evidence codes. We further use protein interactions from the STRING database [21] and protein and genetic interactions from the BioGRID database [22] to evaluate the results. Figure 2 shows the results of the ROC analysis for predicting genetic interactions, Figure 3 shows the results of the ROC analysis for predicting PPIs (extracted from GO annotations), Figure 4 shows the results of the ROC analysis for predicting PPIs from STRING and Figure 5 shows the results of the ROC analysis for predicting interactions from the BioGRID database. We find that the performance of predicting genetic interactions and PPIs based on gene functions improves for every species when including the gene functions we infer. The results are summarized in Table 4, and detailed evaluation results are provided as Supplement S2.

**Table 4:** Summary of results, including the number of inferred functions and the 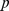 value resulting from a one-tailed t-test comparing the ROC AUC for predicting genetic interactions using the original functional annotations vs. the combination of original and inferred annotations.

Species	Number of novel inferred functions	Number of known inferred functions	BioGRID: ROC AUC (GOA)/ROC AUC (GOA+inferred functions)	STRING-PPI: ROC AUC (GOA)/ROC AUC (GOA+inferred functions)	PPI: ROC AUC (GOA)/ROC AUC (GOA+inferred functions)	GI: ROC AUC (GOA)/ROC AUC (GOA+inferred functions)	p-value (BioGRID)	p-value (STRING-PPI)	p-value (PPI)	p-value (GI)
Fish	1,409	679	0.835/0.915	0.717/0.738	0.835/0.889	0.902/0.952	0.204	0.044	0.218	5.1×10
Yeast	12,483	1,450	0.664/0.686	0.721/0.723	0.892/0.895	0.894/0.896	<10^−6^	0.130	0.243	0.334
Fly	1,057	517	0.764/0.799	0.747/0.749	0.821/0.822	0.854/0.870	4.7 × 10^−4^	0.357	0.475	0.088
Worm	3,885	5,336	0.763/0.791	0.801/0.814	0.794/0.828	0.806/0.851	3.1 × 10^−4^	4.8 × 10^−5^	5.5 × 10^−3^	8.7 × 10^−5^
Mouse	14,013	2,135	0.807/0.832	0.771/0.791	0.853/0.867	0.925/0.935	0.223	<10^−6^	9.2 × 10^−3^	5.9 × 10^−3^
Total	32,847	10,108	0.665/0.687	0.745/0.754	0.867/0.877	0.896/0.907	<10^−6^	<10^−6^	1.4 × 10^−3^	8.2 × 10^−5^

One example of our evaluation is provided by *Casp1* (caspase 1, MGI:96544) and *Il1b* (interleukin 1 beta, MGI:96543), which are known to interact in mice and both are essential for several shared functions [24]. Based on the asserted GO functions, their functional similarity is relatively low. However, based on the phenotypes observed for caspase 1 mutations and interleukin 1 beta mutations, we infer several new functions in which both are involved, including *defense response to bacterium* and *interleukin-1 beta secretion*. We also infer the involvement of *Casp1* in *inflammatory response* which is a known function of *Il1b*. As a consequence of the novel functional annotations, *Casp1* and *Il1b* are functionally significantly more similar than currently inferred through asserted functional annotations (full data provided as Supplement S2).

### Comparison to related work

The most similar related work of which we are aware are explicit mappings between phenotype terms and GO terms that have been created as part of the curation pipeline in WormBase (available at http://wiki.wormbase.org/index.php/Gene_Ontology#Phenotype2GO_pipeline_.28Sanger_and_Caltech.29). In these mappings, particular PATO-based ontology terms are explicitly and manually mapped to GO terms that can reliably be inferred based on the phenotype annotation. For example, the WormBase Phenotype Ontology term *Long* (WBPhenotype:0000022) is mapped to the GO term *negative regulation of multicellular organism growth* (GO:0040015), and these mappings are used to infer functional annotations from mutant phenotype automatically in WormBase. In our approach, we use the PATO-based definitions that have been created for the WormBase Phenotype Ontology [25] to infer gene functions, which leads to complementary functional annotations. In particular, as a consequence of automated inference of GO functional annotations from phenotypes in WormBase, we observe the highest overlap of functions we infer with existing GO annotations (see Table 4), while we nevertheless infer a large number of novel functions that cannot currently be identified through WormBase’s mappings. Furthermore, we use an ontology-based approach to extend such a mapping between observed phenotypes and functional annotations to other model organism species. In particular, we reuse the large number of PATO-based definitions [7] for phenotype ontologies that has recently been created [8], [26], and are therefore able to apply our approach to any model organism data for which such definitions have been created. Furthermore, model organism databases such as ZFIN [11] use PATO-based phenotype descriptions directly, and our method is directly applicable to such phenotypes.

There are a large number of automated function prediction algorithms that utilize text mining [4], [27]–[29], interaction networks [5] and sequence information [6]. Our approach incorporates experimentally derived phenotype data that has, to the best of our knowledge, not yet been incorporated on a large scale into GO function prediction algorithms.

### Electronically Inferred Annotation and “Downstream” Effects

While our approach will not replace the experimental validation and manual curation of functional information in model organism databases, it is, to the best of our knowledge, the first large-scale approach to infer gene functions from phenotype information. With the emergence of genome-wide phenotyping projects, our method provides the necessary tool to bridge the gap between the availability of phenotype information and the inference of functions. In particular, traditional literature curation alone will not be applicable to the analysis of phenotypes resulting from high-throughput phenotyping efforts and the insights they can provide into gene functions, primarily since they are not directly reported in literature.

Our method electronically infers functions from mutant phenotypes and will not create GO annotations in which scientists can have the same confidence as in manually created annotations. However, we have demonstrated the great utility of inferring some annotations electronically from experimental data, in particular the improvements these novel annotations can bring to computational analyses and the prediction of genetic interactions and PPIs. GO evidence codes are used to indicate the source and evidence for an annotations, and our annotations would obtain an *inferred by electronic annotation* (IEA) evidence code. An evidence code specifically indicating that the electronic annotation was made based on the analysis of mutant phenotypes would further improve the accuracy of the evidence code annotation.

One limitation of our approach is its inability to distinguish between direct involvement of a gene in a biological process or function, and the involvement of a gene through regulation of other genes, or functions, that are directly responsible for the resulting phenotypes. This phenomenon, known as “downstream effects” (cf. http://www.geneontology.org/GO.annotation.conventions.shtml#Downstream_Process_guidelines), is a major concern for GO annotations. Currently accepted practices resolve this issue by requesting more specific terms to be added to GO and annotating to these terms instead. In particular, parthood and regulatory terms, which are defined using appropriate *part-of* or *regulates* relations, should be used instead of annotating to the more general process in which genes are only involved indirectly. By following the relations used in defining the more specific terms, involvement in the general process can then be defined based on the GO structure [1]. As our inference of GO functions is based on phenotype information alone, we cannot infer the specific function in most cases. Often, additional experiments would be required to determine how a gene leads to an observed phenotype. In some cases our annotations will be rated high-level, but nevertheless are likely to be useful and correspond to GO annotations that can be inferred if the specific function of the gene was known, assuming the appropriate relations between processes and functions are asserted in GO and the phenotype annotations and the definition of phenotype terms are correct. Our manual analysis of annotations to processes in erythrocyte development and differentiation, however, suggests that in some cases we are able to suggest more specific annotation based on underlying experimental phenotype data.

### Relevance for Scientific Analyses

One of the most widely adopted applications of GO-based gene function annotation falls in the domain of analysis and interpretation of gene expression data [30]. This method relies on the quality and quantity of available functional annotation of genes and gene products, and our method has the potential to further improve the accuracy and statistical power of such analyses. Gene functions are also widely used to infer relations between genes and gene products, including the construction of genetic and protein interaction networks [31], the identification of causal genes in diseases [32] or for drug discovery and drug repurposing [33]. All these approaches can be improved with a higher coverage of reliable functional gene annotations, and further extend the functional analysis of gene expression datasets using data observed in phenotype experiments.

A further computational application for functional annotations is the prediction of genetic interactions and PPIs, and we have demonstrated that both tasks improve significantly when the gene functions we infer are included. This improvement is measurable even when the electronically inferred annotations currently available for genes are taken into consideration, thereby demonstrating that our approach is complementary to other electronic annotation methods.

However, we find significant differences between species when predicting genetic interactions and PPIs. For example, we observe only a small increase in ROC AUC for yeast, although we infer a large number of novel gene functions, while we observe a high increase in ROC AUC for predicting both genetic interactions and PPI in zebrafish, although the number of gene functions we infer is much lower. One explanation for this observation may be the different completeness of annotations in different species, either as a result of different cost and complexity of functional genomics experiments (which is lower in yeast than for most other species), or as a consequence of different resources available for annotating gene functions in the various model organism databases. Furthermore, our evaluation datasets contain large differences in the number of interactions within each species. We aim to account for these divergent numbers of positive and negative examples of interactions by using a t-test to compare the difference in ROC AUC. Nevertheless, the ROC AUCs reported for species with low numbers of known interactions will be less accurate than ROC AUCs for species with a high number of known interactions, and this may explain parts of the differences observed in ROC AUC.

### Future Research

Currently, we are conservative in the assumptions we make that allow us to infer functional information. However, our approach can be extended to infer more detailed and complex functional information. For example, if an abnormal *morphology* of the tail is observed as a phenotype resulting from a mutation in a gene, then this gene will likely be involved in *tail morphogenesis*. However, in some cases such a phenotype may not immediately be the consequence of mutations in the gene but rather the result of an impaired function of another gene that is related with the mutated gene through a biochemical, cellular or physiological pathway. In future research, an explicit representation of such interactions, in particular on an organism-wide physiological scale, will further improve the performance of our method.

## Supporting Information

Supplement S1 **Inferred GO functions.** A complete dataset of inferred GO annotations from phenotypes. Each file contains the gene idenfier and the novel GO functions we infer for each species.
(ZIP)

Supplement S2 **Computational evaluation results.** A complete dataset for predicting interactions (genetic interactions, PPIs from GO, PPIs from STRING and interactions from BioGRID) using GO functional similarity. The first two columns of each file contains the interaction partners, the third column contains the position of the interaction pair in the functional similarity list (i.e., a value of 0 indicates that both partners are the functionally most similar, while a value of 1 indicates that both partners are the functionally least similar) based on GOA annotations, and the fourth column indicates the position of the interaction pair in the functional similarity list based on GOA’s and our inferred annotations.
(BZ2)

Supplement S3 **Manual evaluation results.** A dataset of manually evaluated inferred functions. The file contains an inferred function for 20 genes from yeast, worm, fruitfly, zebrafish and mouse, as well as a PubMed reference to a manuscript providing evidence for the function.
(ZIP)
